# Overview of Celiac Disease in Russia: Regional Data and Estimated Prevalence

**DOI:** 10.1155/2017/2314813

**Published:** 2017-02-20

**Authors:** Lyudmila V. Savvateeva, Svetlana I. Erdes, Anton S. Antishin, Andrey A. Zamyatnin

**Affiliations:** ^1^Institute of Molecular Medicine, Sechenov First Moscow State Medical University, Moscow 119991, Russia; ^2^Faculty of Pediatrics, Sechenov First Moscow State Medical University, Moscow 119991, Russia; ^3^Belozersky Institute of Physico-Chemical Biology, Lomonosov Moscow State University, Moscow 119992, Russia

## Abstract

Celiac disease (CD) is an autoimmune enteropathy triggered by the ingestion of dietary gluten from some cereals mainly in individuals carrying the HLA-DQ2 and/or HLA-DQ8 haplotypes. As an autoimmune disease, CD is manifested in the small intestine in the form of a progressive and reversible inflammatory lesion due to immune response to self-antigens. Indeed, CD is one of the most challenging medicosocial problems in current gastroenterology. At present, the global CD prevalence is estimated at approximately 1% based on data sent from different locations and available CD screening strategies used. However, it is impossible to estimate global CD prevalence without all the data from the world, including Russia. In this review, we summarize the data on the incidence and prevalence of CD across geographically distinct regions of Russia, which are mostly present in local Russian scientific sources. Our conclusion is that the situation of CD prevalence in Russia is higher than is commonly believed and follows global tendencies that correspond to the epidemiologic situation in Europe, America, and Southwest Asia.

## 1. Introduction

Celiac disease (CD) is an autoimmune-based disorder triggered by ingested gluten-containing grains in genetically predisposed individuals [1]. CD patients have a wide range of both gastrointestinal (chronic diarrhea with weight loss and malabsorption) and nonintestinal (e.g., iron-deficiency anemia, osteoporosis, and “autoimmune” conditions) symptoms revealing typical and atypical forms of CD. CD can be identified at any age and in both genders or stay undiagnosed. In recent years, considerable changes in the epidemiology of worldwide CD have been observed, and the development of proper screening tests has led to estimations of its incidence.

CD pathogenesis involves the adaptive (HLA molecules, transglutaminase 2, dendritic cells, and CD4(+) T-cells) and the innate immunity with an IL-15-mediated response elicited in the intraepithelial compartment. Currently, the only treatment is a permanent strict gluten-free diet (GFD) [2]. However, some cereals are an important source of proteins, lipids, vitamins, minerals, and fibre, and their inclusion in a gluten-free diet might improve the nutritional status of celiac patients but the immunogenicity of certain grain cultivars should be thoroughly tested [3, 4]. Since CD is a common and lifelong disorder, many studies have focused on the intensive development of drugs for the treatment of gluten intolerance [5]. Medical treatment will significantly improve the quality of life of patients with gluten-related diseases by allowing a nonstrict gluten-free diet.

Since CD is the result of both environmental and genetic factors the world population distribution of CD is determined by gluten consumption and mainly class II human leukocyte antigens (HLA) genes (HLA-DQ2, HLA-DQ8). On this basis, it is evident that gluten intolerance is more common in Europe, South and North Americas, Australia, Southwest Asia, and North Africa and less common in the Far East. This is confirmed by the recent increase in large-scale population-based studies (see Lionetti et al. [6] for a review). Research indicates that the CD prevalence is approximately 1% of the general population, with variations being due to the high rate of hidden and atypical forms of the disease.

**Figure 1:**
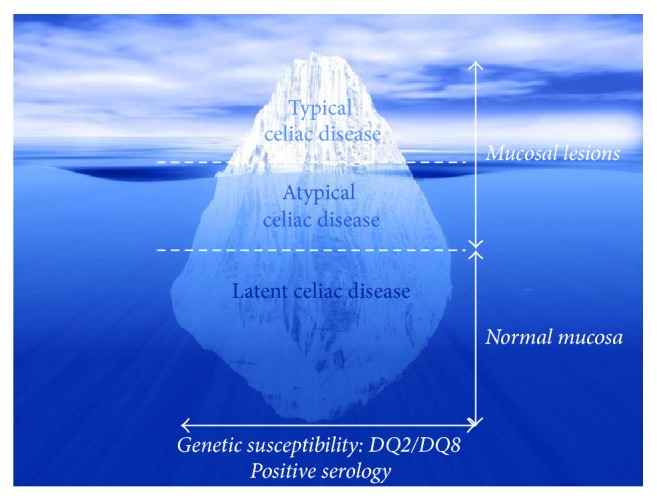
The celiac iceberg (adapted from Catassi et al. [8] and Fasano and Catassi [9]).

In general, the display of epidemiological changes of CD can be represented by the “iceberg” model, originally proposed by Logan [7] and later promoted by Catassi and Fasano (Figure 1) [8, 9]. Thus, it was suggested that the total size of the “iceberg” is more or less the same worldwide, although the “waterline” (the ratio of diagnosed to undiagnosed cases) may dynamically shift depending on the region and population as well as clinician awareness, availability of diagnostic tools, and the degree of clinical manifestations of the disease and so forth [9]. This clarifies some divergences in the results of discrete population studies.

The worldwide distribution of CD cannot be reliable without data from Russia, which occupies one-sixth of the global land area. Thus, the aim of this review is to present the data available on CD epidemiology in Russia and to generate preliminary insight into this common chronic enteropathy.

## 2. CD Diagnosis and Screening Strategies

CD can be diagnosed according to the current guidelines following certain criteria, for example, updated algorithms of (i) the European Society for Pediatric Gastroenterology, Hepatology and Nutrition (ESPHGAN), (ii) North American Society for Pediatric Gastroenterology, Hepatology and Nutrition (NASPGHAN), (iii) World Gastroenterology Organization (WGO-OMGE), (iv) British Society of Gastroenterology, and (v) American College of Gastroenterology [10–13]. Updated algorithms following major global guidance were also recommended for use in Russia [14]. The progress in the development of immunobiological and genetic laboratory tests screening associated with CD and gastrointestinal endoscopic techniques led to an increased CD frequency detection in patients with both typical and atypical (asymptomatic) clinical courses. Besides, it is possible to detect the disease in at-risk groups, which is comprised of persons with genetic predisposition, relatives of CD patients, people with iron-deficiency anemia, premature osteoporosis and osteopenia, type 1 diabetes mellitus, autoimmune thyroid disease, liver disease, Down syndrome, or Turner's syndrome, and patients suffering from some other diseases [15]. Some researchers suggest that the timely detection of CD in at-risk groups and subsequent treatment can reduce the severity of CD complications [16, 17].

Genetic susceptibility in CD is linked to HLA genes DQ2 and DQ8, namely, HLA-DQA1^*∗*^05-DQB1^*∗*^02 (DQ2) and DQA1^*∗*^03-DQB1^*∗*^0302 (DQ8) [18]. The identification of additional disease genes is a research subject for the celiac genetic community. It has been revealed that CD is associated with genes of non-HLA-region [19, 20]; however, it is still believed that CD is unlikely in the absence of the alleles encoding the DQ2 and DQ8 [21].

Screening for CD signs is provided by the use of serologic antibody-based tests (Table 1), which are in most enzyme-linked immunosorbent assays (ELISA). Currently, the optimal diagnostic kits for CD are IgA anti-tTG2- (IgA anti-tissue transglutaminase 2-) based ELISA and IgA AEA- (IgA anti-endomysial antibodies-) based IFA (detected by indirect immunofluorescence assay) due to their high sensitivity and specificity and reliability [22]. However, some studies described below still use outdated methodology for the serologic detection of anti-gliadin antibodies (IgA AGA, IgG AGA). This methodology is not currently recommended for CD diagnosis due to the low positive predictive value compared to other available serological tests [22].

The values of serological antibody screening tests have long been recognized in preselecting patients for small intestinal biopsy diagnosis. Intestinal biopsy is considered as the “gold standard” for the diagnosis of CD. For instance, small-bowel mucosal villous atrophy and crypt hyperplasia detection remain a central test in CD diagnosis (Table 1), and an increase of intraepithelial lymphocytes may indicate latent CD [15]. The grading of morphological alternations according to the Marsh-Oberhuber classification is widely used in Russia to diagnose CD [23].

Decisions on screening for CD should be carefully considered. Persons from at-risk groups are usually involved in these studies (case-finding process). However, this strategy is ineffective for detecting undiagnosed CD in the wider population. In contrast, mass screening is extremely expensive and depends on a number of assumptions. Thus, it is considered that efficient screening CD programmes might be carried out by testing school-age children [24].

## 3. Present Epidemiologic Data on CD in Russia

Until recently, celiac disease in Russia has been considered as a rare disease with a frequency of 1 to 5 per 10,000 persons, but, as elsewhere, the advance in CD diagnosis has changed this, leading to the identification of various forms of CD (or gluten intolerance). Thus, the growing incidence rate, when compared with the population of the whole region, has revealed that CD prevalence in Russia has increased from about 0.02% to 0.30% (Table 2).

**Figure 2:**
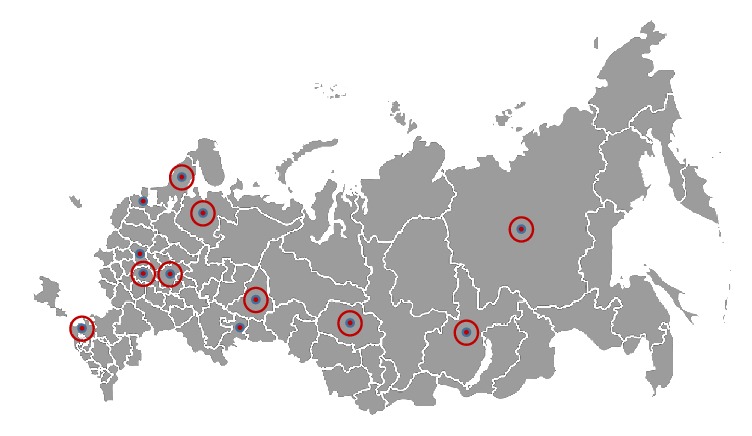
Geographic distribution of incidence reports and prevalence studies on CD in Russia. Small circles indicate that the data were only obtained from the regional center. Rounded circles indicate that the data were collected from several locations within the region. See Tables 2 and 3 for the results of these studies.

Massive studies on CD prevalence in Russia have not yet been carried out, and the only detailed study presented in the international scientific literature was focused on CD prevalence in Karelia [25]. In this study, the frequency of biopsy-proven CD was reported as 1 : 496. In addition, a number of local publications can be found [26, 27], which are only available in Russian. These reports contain scattered data from different regions of Russia (Figure 2), with the CD prevalence varying from 0.20% to 0.57% in the general population and up to 15.98% in specific risk patient groups (patients from specialized gastrointestinal clinics who are suffering from chronic diarrhea, other manifestations of enteropathy, iron-deficiency anemia of unknown genesis, type 1 diabetes mellitus, autoimmune thyroid disease, etc.) (Table 3).

Epidemiological studies were also conducted in two CIS (Commonwealth of Independent States) countries: Kazakhstan and Uzbekistan. Thus in Almaty, Kazakhstan (as a “reflection” of the demographic situation in Kazakhstan, in general), CD prevalence among children was found as 1 : 262 (0.38%) with the 1 : 5 ratio of typical to atypical CD forms [28]. According to the Pediatric Research Institute of the Republic of Uzbekistan (Tashkent region) the CD incidence was 1 : 366 (0.27%) [29].

Studies on polymorphic variants of CD susceptibility alleles HLA-DQ2 and/or HLA-DQ8 in CD patients using HLA SSP typing have been conducted in several regions of Russia (75 subjects in Tomsk, 32 subjects in Krasnodar, and 17 subjects in Yakutia [30–32]) and in Kazakhstan (72 CD patients in Almaty [28]). These studies report that 76.9% CD patients in Tomsk, 81.2% CD patients in Krasnodar, and 80.9% CD patients in Yakutia carry HLA-DQ2 and/or HLA-DQ8 [30–32]. Thus, in CD patients from Tomsk and Krasnodar the presence of 8 allelic variants for HLA-DQA1 loci and 12 allelic variants for HLA-DQB1 loci was screened. The most frequent alleles detected among CD patients in Krasnodar were DQA1^*∗*^0501 (40.6%), DQA1^*∗*^0201 (21.9%), and DQ*В*1^*∗*^0201 (35.9%), whereas the most frequent allele reported for patients from Tomsk was DQA1^*∗*^0501 (37.3%) [30, 31]. Three-locus HLA haplotype screening (DRB1, DQA1, and DQB1) was performed in CD patients from Yakutia. In this study, the following haplotype frequencies were detected: 30% for DRB1^*∗*^04/DQA1^*∗*^0301/DQB1^*∗*^0302, 30% for DRB1^*∗*^03/DQA1^*∗*^0501/DQB1^*∗*^0201, 25% for DRB1^*∗*^07/DQA1^*∗*^0201/DQB1^*∗*^0202, and 15% for DRB1^*∗*^11/DQA1^*∗*^0505/DQB1^*∗*^0301 [32]. In Kazakhstan the presence of HLA-DQ2 and/or HLA-DQ8 was identified even less frequently then in the regions of Russia and was found only in 60.2% of the samples obtained from CD patients [28]. Thus, the screening of CD susceptibility HLA alleles in Kazakh patients showed the following haplotype frequencies: 26.4% for DQA1^*∗*^0501 and DQB1^*∗*^0201, 34.7% for DQA1^*∗*^0501 or DQB1^*∗*^0201, and 8.3% for DQA1^*∗*^0301 and DQB1^*∗*^0302. Furthermore, the authors suggest pointing special attention on the availability of DRB1^*∗*^10 allele in CD patients, the frequency of which was detected as 15.3% [28].

## 4. Discussion

In addition to the one detailed study focusing on the CD prevalence in Russia that has been published in the international scientific literature [25], several reports from geographically distinct regions on the topic can be found in local Russian scientific literature (summarized in Tables 2 and 3). These studies include reports on incidence rate and reports on CD screenings, most of which were performed in specific risk groups. However, the design of these studies and arsenal of diagnostic tools used for CD diagnosis, in some cases, varies considerably, and this complicates the comparison of reported results. Nonetheless, these studies do show that the preliminary general situation of CD prevalence in Russia seems to follow the general global tendencies.

CD is often diagnosed in the first year of life in children after introducing gluten-containing cereals into the mixed feeding. According to the recommendations of the Pediatric Union of Russian Federation it is preferable to introduce mixed feeding and in particular cereals at the age of 4–6 months. It is recommended to start a mixed feeding diet with the addition of gluten-free cereals such as rice and buckwheat, which is very popular in the Russian population, and later with maize. Then gluten-containing cereals can be included [33]. Thus, the first CD symptoms can be detected during early childhood. Indeed, all the data on CD incidence rates per Russian region is represented by children's cases (Table 2). Moreover, a considerable part of the studies that focus on CD prevalence in Russia were obtained from children (Table 3). Comparable data on CD prevalence in children and in adults only exists for the Irkutsk region, where a higher CD prevalence was reported in children than in adults (Table 3) [34]. Environmental factors influencing infancy or CD latency in adulthood can explain this fact [35].

The data on the incidence rate per region population (Table 2) shows that CD prevalence in Russia is increasing by up to 0.3%. Nonetheless, it is obvious that this is an underestimation and not a true reflection of the incidence of the disease. Thus, the screening data on CD prevalence in schoolchildren and healthy blood donors studied in Russia varies by up to 0.6% (Table 3). These results follow the global tendencies presented in the literature for these categories of cohorts studied in various countries of Europe, America, and Southwest Asia [36–38].

CD prevalence among the population of the specific risk groups studied in Russia varied from approximately 1% up to 16% (Table 3). Unfortunately, the recruitment procedures for the patients involved in these studies were too briefly described in the original papers, which makes the comparison of the results with other studies even more difficult. Nonetheless, these results are comparable with the results from a number of studies that focus on the assessment of CD prevalence in different risk groups from distinct countries reporting CD prevalence for risk groups, which are 2–10 fold in comparison to the CD prevalence found among the entire populations [36, 39, 40].

Results on CD incidence (Table 2) and prevalence in the different regions of Russia obtained for children cohorts (Table 3) revealed no clearly differentiated gender-related dependence. These data are in contradiction with well-documented general female dominance among CD patients [41] and can be explained by the peculiarities of the patients' recruitment among the children falling into the specific risk groups (Table 3). Nonetheless, the higher prevalence of CD in adult women was registered (Table 3), which is in accordance with other studies from other locations [42, 43]. Female dominance among CD patients is usually explained by the deficiency in healthcare services or hormonal differences between genders [42, 43]. Conversely, the higher prevalence of CD in men among blood donors can be explained by the bias towards men in the cohort of subjects involved in the study [44].

To date, studies on polymorphic variants of HLA DQA1 and DQB1 genes in CD patients have only been performed in three regions in Russia (Tomsk, Krasnodar, and Yakutia) [30–32]. Indeed, genotypes of CD patients in different regions have their own characteristics and the absence of certain alleles does not exclude the possibility of the development of the disease. It was found that the incidence of susceptibility alleles HLA-DQ2 and/or HLA-DQ8 in several regions of Russia is significantly lower than in Europe (approximately 80% versus >90%) [45, 46]. Remarkably, in Kazakhstan the frequency of these susceptibility alleles in CD patients was reported as being even lower than in mentioned regions of Russia (approximately 60%) [28]. It is well-documented that frequency of susceptibility alleles in Caucasian populations in Western Europe has been estimated at 20–30%. Relatively high frequencies also occur in Northern and Western Africa, the Middle East, and central Asia, whereas high frequencies of susceptibility alleles decline from West to East with low frequencies in populations in Southeast Asia and the virtual absence of DQ2 in Japan [47]. Thus, one could speculate that the lower frequency of these alleles in the patients from several regions of Russia and, especially, in patients from Kazakhstan is a result of the lower frequency of these alleles in the general populations of these regions.

The haplotypes of genetic susceptibility to CD HLA-DQ2 (DQA1^*∗*^05, DQB1^*∗*^02) found in patients from Russian regions [30, 31] are consistent with those in Europe [48]. Relatively high frequencies for HLA-DQ8 (DQA1^*∗*^03, DQB1^*∗*^03) (~30%) detected in patients from Yakutia [32] can be explained by the low number of patients involved in the study and/or by the specific features of the study, in which both Caucasians and Mongoloid subjects were recruited. Nevertheless, due to the low number of patients involved in the studies in Russia the incidence of polymorphic variants of HLA DQA1 and DQB1 genes in CD patients needs to be studied further.

One could speculate that, by comparing the results from one Russian region (Karelia; 0.2%) with Finland (0.9%), the low CD prevalence in Russia is explainable by the poorer living conditions and hygiene standard [25]. However, the comparison of available data on the average frequency of CD distribution in Russia (Table 3), with its distribution in European countries (e.g., 0.3% in Germany, 0.7% in Italy, and 1.8% in Sweden [49, 50]), shows comparable results, which makes such a speculation unlikely.

## 5. Support for CD Patients in Russia

Celiac patients in Russia are registered as chronic patients and undergo medical checkups once every 6 months (after diagnosis within the first two years) or 12 months (the third year of observation subject to the establishment of stable remission). On a Federal level, there is social support (free set of gluten-free products) for children diagnosed with “celiac disease” [51]. In some regions, children with CD (but without a status of disabled person) receive social support from the regional governments (monthly grants) [52].

In addition, in Russia few local associations exist for patients with celiac disease designed to implement different projects aimed at social support and improving the quality of life of people following a strict gluten-free diet (GFD). They keep web pages that disseminate information about CD and various events for CD patients, with people needing to follow GFD [53–55].

Gluten-free products may be purchased in specialized shops, e-shops, or some supermarkets, and the list of gluten-free products is gradually expanding. Novel gluten detoxification enzymatic tools are under development [56].

## 6. Conclusions

The present data on CD prevalence in Russia may give us some notion about the gluten intolerance situation in the country. As shown the average CD frequency in the general Russian population is about 0.2–0.6%. However, the real rate is still unknown due to the absence of true diagnostic data from all the citizens. The presented Russian CD prevalence values can be well-explained by the “celiac iceberg” concept, and, for this reason, we believe that large-scale diagnostic screening covering all the major regions of Russia following certain criteria will clarify the true situation about gluten-related diseases and, in particular, CD.

## Figures and Tables

**Table 1:** CD screening (in patients with manifestation of clinical symptoms).

	Serology (serologic markers)^*∗*^	Endoscopy of duodenum (mucosal morphologic alternations^*∗∗*^)	Genotype (genetic markers)
	IgA anti-tissue transglutaminase antibodies (IgA anti-tTG, IgG anti-tTG antibodies)	Mucous membrane thickness	HLA-typing
	Anti-endomysial antibodies (AEA, IgA-anti-EMA)	Villus height	
	Anti-deamidated gliadin peptide (IgA anti-DGP, IgG anti-DGP)	Crypt depth	
		Crypt-villus ratio	

*Limitations*	Specificity and sensitivity of test method; the active period of the disease; long-term gluten-free diet	Similar histological pattern of some other diseases; long-term gluten-free diet	—

^*∗*^Some studies described in the current review still used outdated methodology for the serologic detection of IgA anti-gliadin antibodies (IgA AGA, IgG AGA).

^*∗∗*^These criteria are particularly used for the diagnosis of CD, according to Marsh-Oberhuber classification [23].

**Table 2:** CD incidence rate per the region population.

Region	Year	Age group	Sex ratio (m%/f %)	Screening test	Biopsy	CD (%)	Author
Arkhangelsk region	2005	Children (6 mths–18 yrs; <7 yrs, 54%)	60.0/40.0	Anti-tTG and/or AEA	Yes	0.02%	Smirnova et al. [57]
Chelyabinsk	2004	Children (0–18 yrs)	ND	AGA and/or anti-tTG and/or AEA	Yes	0.02%	Turchina and Tabak [58]
Sakha Republic (Yakutia)	2008	Children (6 mths–18 yrs)	48.4/51.6	Anti-tTG	Yes	0.06%	Savvina et al. [59]
Saint Petersburg	1999–2002	Children (6 mths–18 yrs)	50.0/50.0	Anti-tTG and/or AEA	Yes	0.02%	Vasilkova [60]
Sverdlovsk region	2009	Children (0–18 yrs)	ND	AGA and/or anti-tTG and/or AEA	Yes	0.30%	Klimin et al. [61]
Tomsk region	2010	Children (mean age 8.6 ± 0.6)	50.5/49.5	Anti-tTG and/or AEA	Yes	0.05%	Yankina [62]
Yakutsk	2008	Children (6 mths–17 yrs; <4 yrs, 8.7%; 5–7 yrs, 28.8%; 8–10 yrs, 25.0%; 11–14 yrs, 26.3%; 15–17 yrs, 11.2%)	65.0/35.0	Anti-tTG and/or AEA	Yes	0.11%	Savvina et al. [63]

ND: not determined.

**Table 3:** Screening results of CD prevalence obtained from geographically distinct regions of Russia.

Region	Year	Participants	Age group	Sex ratio (m%/f %)	Screening test	Number screened	Screen positive	Biopsy	Number biopsied	CD	Author
Karelia region	1997–2001	Schoolchildren	Children (mean age 11.6 ± 6.7)	49.5/51.5	Anti-tTG	1988	8	Yes	4	1 : 496 (0.20%)	Kondrashova et al. [25]
Ryazan region	ND	Healthy blood donors	Adults	69.9/30.1	Anti-tTG	1740	41	Yes	10	1 : 174 (0.57%)	Stroikova et al. [44]

Irkutsk region	2011	Specific risk^*∗*^	Children (6 mths–18 yrs)	52.0/48.0	Anti-tTG	1441	390	Yes	60	1 : 18 (5.56%)	Reshetnik et al. [34]
			Adults	33.4/66.6		1402	271	Yes	45	1 : 31 (3.32%)	
	2014	Specific risk^*∗*^	Children (6 mths–18 yrs)	51.9/48.1		1775	494	Yes	12	1 : 40 (2.50%)	Reshetnik et al. [64]
Krasnodar region	2010	Specific risk^*∗*^	Children (6 mths–18 yrs)	46.9/53.1	Anti-tTG and/or AEA	742	54	Yes	54	1 : 36.6 (2.73%)	Tlif et al. [30]
Moscow	2003–2007	Specific risk^*∗*^	Adults	19.8/80.2	Anti-tTG	363	58	Yes	58	1 : 6.2 (15.98%)	Gudkova et al. [65]
	2012	Specific risk^*∗∗*^	Adults (mean age 51.5 ± 16.4)	ND		318	6	Yes	5	1 : 106 (0.94%)	Bykova et al. [66]
Nizhny Novgorod region	2008	Specific risk^*∗*^	Adults	ND	Anti-tTG	1045	251	Yes	311	1 : 7.6 (13.16%)	Repin et al. [67]
Ryazan region	2002–2006	Specific risk^*∗∗∗*^	Children (6 mths–18 yrs)	57.4/42.6	Anti-tTG	256	4	Yes	4	1 : 57 (1.75%)	Stroikova et al. [68]

^*∗*^Patients with symptoms suggestive of CD: malabsorption syndrome, chronic abdominal pain, cramping or distension, chronic or intermittent diarrhea, growth failure, iron-deficiency anemia, weight loss, chronic fatigue, short stature, recurrent aphthous stomatitis (mouth ulcers), dermatitis herpetiformis-type rash, atopic dermatitis, urticaria, vitiligo, bronchial asthma, and so forth.

^*∗∗*^Participants were recruited among patients from specialized gastrointestinal clinics.

^*∗∗∗*^Patients with symptoms suggestive of CD (chronic or intermittent diarrhea, failure to thrive, weight loss, stunted growth, delayed puberty, amenorrhea, iron-deficiency anemia, chronic abdominal pain, cramping or distension, chronic fatigue, recurrent aphthous stomatitis (mouth ulcers), dermatitis herpetiformis-like rash, and atopic dermatitis) and asymptomatic patients at increased risk for CD (type 1 diabetes mellitus, Down syndrome, autoimmune thyroid disease, and first-degree relatives with CD).

ND: not determined.
